# Influenza Vaccine Effectiveness in Korea: A Systematic Review and Meta-Analysis of Real-World Evidence from the Past Decade

**DOI:** 10.3390/vaccines14030217

**Published:** 2026-02-27

**Authors:** Hye Su Jeong, Hye Young Kim

**Affiliations:** Medical Affairs, SK bioscience, 38, Yeongudanji-ro, Yeonsu-gu, Incheon 21984, Republic of Korea; hyesujeong@sk.com

**Keywords:** influenza vaccines, vaccine effectiveness, meta-analysis, Republic of Korea

## Abstract

Background: Influenza remains a significant public health burden. Although vaccination is the most effective preventive strategy, evidence on influenza vaccine effectiveness (VE) in Korea remains fragmented. Methods: A systematic review and meta-analysis were conducted on real-world Korean influenza VE studies published from January 2016 to October 2025. Literature research was performed in PubMed, Embase, and Web of Science. Eligible studies evaluated influenza VE using real-world data sources. Pooled VE estimates were calculated using a random-effects model and stratified by age group and virus type. Results: A total of 2922 records were identified, of which nine studies met the inclusion criteria and eight were included in the meta-analysis. Eight of nine studies were conducted in hospital settings. Five studies targeted adults (including two focusing on those aged ≥ 65 years), and four targeted children. Six of the nine studies evaluated single influenza seasons, while the remaining three covered multiple seasons. Statistically significant VE was observed among adults (23.6%, 95% confidence interval [CI]: 13.8 to 32.2), children (25.2%, 95% CI: 8.2 to 39.0) and against influenza A in children (32.4%, 95% CI: 17.7 to 44.4). Conclusions: This study synthesized a decade of fragmented VE evidence in Korea. Findings suggest suboptimal effectiveness compared to global estimates, highlighting the need for further evaluation of alternative vaccine platforms, including cell-based vaccines, within Korea’s national immunization strategy.

## 1. Background

Influenza is an infectious respiratory illness characterized by typical symptoms such as cough, fever, and nasal congestion [1]. Although most patients improve with supportive care and antiviral treatment, some develop severe complications or experience mortality [2]. According to the World Health Organization (WHO) 2024 update on the global burden of influenza, influenza causes an estimated 3–5 million cases of severe illness and 290,000–650,000 respiratory deaths worldwide each year, underscoring its substantial public health burden [3]. Among high-risk populations, older adults and children are particularly vulnerable to influenza-related complications. Older adults may experience pneumonia [4] or multiorgan failure [5], whereas children are susceptible to neurologic complications such as febrile seizures and influenza-associated encephalopathy [6]. Annual influenza vaccination remains the most effective public health strategy for preventing influenza infection and its severe outcomes and is widely recommended as a primary approach to reducing disease incidence and clinical burden [3].

Recognizing the importance of prevention early on, the Korean government introduced publicly funded influenza vaccination through the National Immunization Program (NIP) for adults aged ≥ 65 years at public health centers in 1997. The NIP subsequently expanded coverage to children aged 6 months to 12 years in January 2018 and to pregnant women in October 2019 [7]. Despite these efforts, influenza continues to exert a significant burden in Korea. A multicenter study reported adult influenza incidence rates of 113.1–220.7, hospitalization rates of 35.5–76.8, and mortality rates of 1.4–3.6 per 100,000 population, with an annual socioeconomic burden of US $156–316 million during 2014–2019 [8]. As of 2025, the NIP provides free influenza vaccination to high-risk groups—including children aged 6 months to 13 years, adults aged ≥ 65 years, and pregnant women—using nine inactivated vaccines: eight egg-based and one cell-based [9,10]. These public health considerations highlight the need for robust evidence evaluating influenza vaccine effectiveness (VE) in Korea.

Real-world evidence (RWE) plays a critical role in assessing the actual effectiveness of influenza vaccines under routine clinical conditions. Compared with controlled trials or single-season evaluations, RWE studies allow broader assessments across diverse populations and multiple influenza seasons [11]. However, existing VE studies from Korea have primarily focused on specific target populations—such as older adults [12,13] or children [14,15,16,17]—or single influenza seasons [15,16,17,18,19,20]. While these studies provide valuable age- and season-specific insights, the absence of a comprehensive synthesis across populations and multiple seasons limits the ability to assess overall VE trends and strain-specific performance at the national level. To address this gap, we conducted a systematic review and meta-analysis of real-world influenza VE studies in Korea over the past decade, integrating evidence across age groups, seasons, and virus types.

## 2. Methods

### 2.1. Search Strategy

The study protocol was registered with PROSPERO (registration number: 1241264). A comprehensive literature search was conducted for studies published from 1 January 2016 to 22 October 2025, covering approximately a decade of data. Searches were performed in PubMed, Embase, and Web of Science. Both Medical Subject Headings (MeSH) terms and free-text keywords were applied, combining influenza, vaccination, and VE. Detailed search strategies were provided in Supplementary Table S1. The review process followed the Preferred Reporting Items for Systematic Reviews and Meta-Analyses (PRISMA) guideline (Supplementary Table S2) [21]. Because only publicly available data were analyzed and no individual human participants were involved, institutional review board approval and informed consent procedures were not required.

### 2.2. Eligibility Criteria and Study Selection

The inclusion criteria were defined before the literature review. Eligible studies met the following conditions: (1) they evaluated influenza VE; (2) they were conducted in Korea; and (3) they utilized real-world data sources, such as electronic medical records, clinical registries, health insurance claims databases, or structured surveys. The exclusion criteria were as follows: (1) studies available only as abstracts, case reports, case series, or review articles; (2) randomized controlled trials (RCTs); and (3) studies not written in English.

### 2.3. Study Identification and Data Extraction

Two independent reviewers (H.S.J. and H.Y.K.) evaluated the titles and abstracts of all retrieved records. Any citation judged as potentially relevant by either reviewer was carried forward to full-text assessment after duplicate entries had been removed. The full articles were then reviewed independently, and differences in judgment were resolved through joint discussion until agreement was achieved. Data extraction was performed separately by both reviewers using a standardized form that was jointly developed through consensus. Information collected from each study comprised the research setting, study population, design, influenza season, diagnostic approach for influenza confirmation, type of vaccine, total number of participants, and the estimated VE. To ensure analytic consistency and avoid duplication of datasets, all extracted information, and references were compared and reconciled between reviewers.

### 2.4. Publication Bias

Publication bias was evaluated using funnel plots and Egger’s test [22]. Egger’s test results indicated no statistically significant evidence of publication bias, with all *p*-values exceeding 0.05.

### 2.5. Assessment of Risk of Bias and Methodological Quality

We evaluated methodological quality and risk of bias using the Risk of Bias in Non-Randomized Studies of Interventions (ROBINS-I) tool [23]. This framework assesses bias across seven domains: confounding, selection of participants, classification of interventions, deviations from intended interventions, missing data, measurement of outcomes, and selective reporting. Two reviewers independently conducted the assessments. Any disagreements were resolved through discussion until consensus was achieved. Each domain was rated as having low, moderate, serious, or critical risk of bias according to the ROBINS-I criteria. Although risk of bias was assessed at the study level, the certainty of evidence for each outcome was not formally evaluated using the GRADE approach.

### 2.6. Analytical Plans

To ensure valid and comparable outcome assessment, only studies with laboratory-confirmed influenza were included in the quantitative analysis; therefore, Kim et al. (2020) [15] was excluded because influenza infection was identified through a survey rather than laboratory testing. VE was defined as 100 × (1 − odds ratio [OR]), comparing the odds of vaccination among laboratory-confirmed influenza cases (test-positive) and controls (test-negative). For studies that reported relative risks, values were converted to the OR scale to maintain consistency across analyses. Because most studies used rapid antigen-based diagnostic tests for influenza (hereafter referred to as RDTs) rather than polymerase chain reaction (PCR) assays for case confirmation, VE estimates from studies that employed both PCR and RDT methods were based on RDT results (e.g., Sohn et al., 2020 [16]). Stratified analyses were conducted where quantitative synthesis was possible, based on age group (adults, elderly, and children), influenza virus type (A and B), and subtype (A/H3N2). A random-effects model was used to estimate the pooled log OR, corresponding 95% confidence interval (CI), and VE [24]. Between-study heterogeneity was assessed using the I^2^ statistic [25].

All statistical analyses were performed using R software (version 4.5.1; R Foundation for Statistical Computing, Vienna, Austria).

## 3. Results

### 3.1. Characteristics of Included Studies

**Figure 1:**
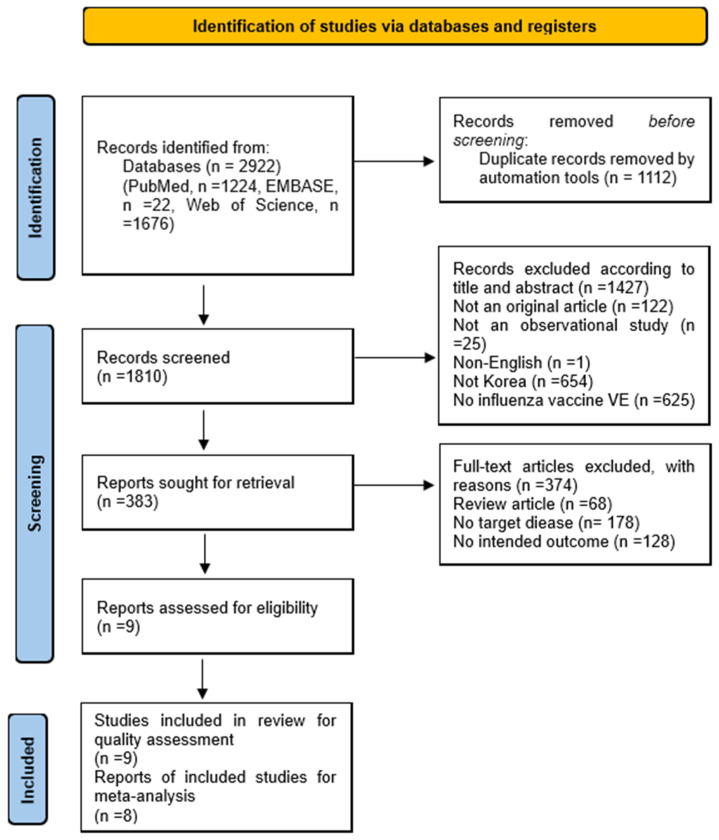
PRISMA flow diagram of study selection.

The literature search identified 2922 records. Following title and abstract screening and duplicate removal, 383 articles underwent full-text review for eligibility. Of these, nine studies satisfied the predefined inclusion criteria [12,13,14,15,16,17,18,19,20], and eight were included in the quantitative synthesis (Figure 1). Eight of the nine included studies were conducted in hospital settings, while one study [15] was based on a school survey. Regarding study design, most studies employed a test-negative design (TND) (n = 7; [12,13,16,17,18,19,20], whereas one study used a cohort design [14] and one employed a cross-sectional design [15]. Among the nine included studies, five targeted adults aged ≥ 19 years [12,13,18,19,20], including two that focused solely on those aged ≥ 65 years [12,13], and four examined children [14,15,16,17]. Six studies were based on a single influenza season [15,16,17,18,19,20], while only three studies included multiple seasons [12,13,14]. All included studies evaluated seasonal influenza vaccines, and sample sizes ranged from 400 to 5322 participants (Table 1).

### 3.2. Findings from Meta-Analysis

**Figure 2:**
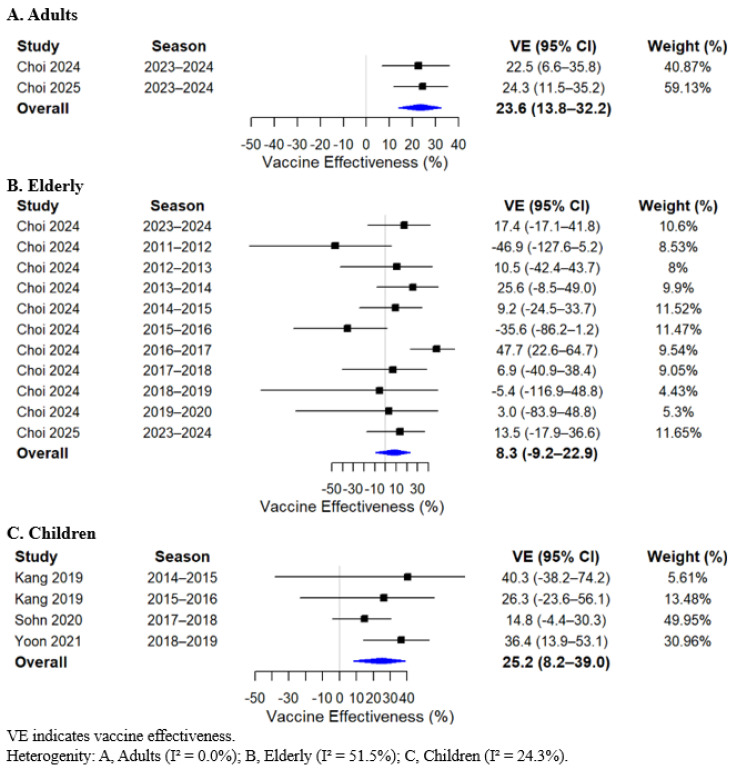
Forest plot of pooled influenza vaccine effectiveness estimates by age group [13,14,16,17,19,20].

**Figure 3:**
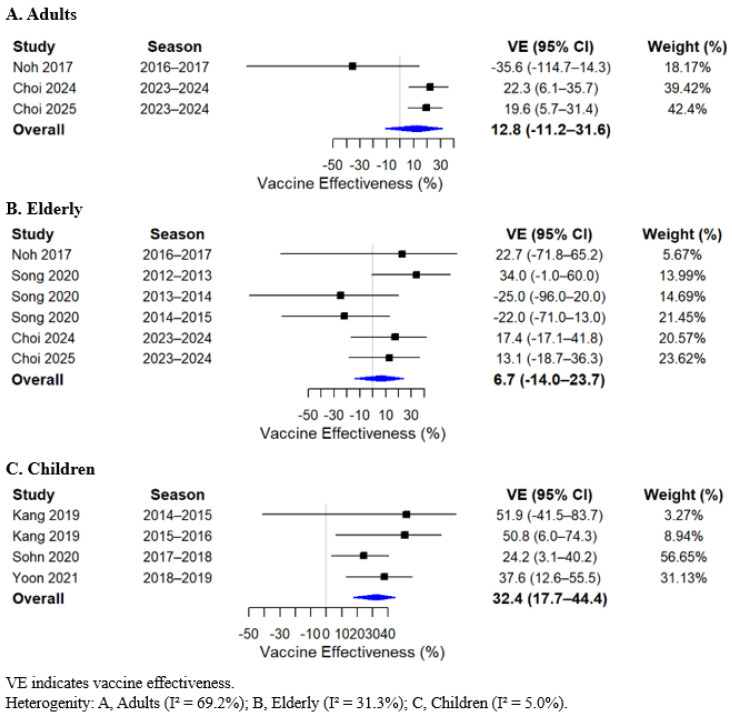
Forest plot of pooled vaccine effectiveness against influenza A by age group [12,14,16,17,18,19,20].

**Figure 4:**
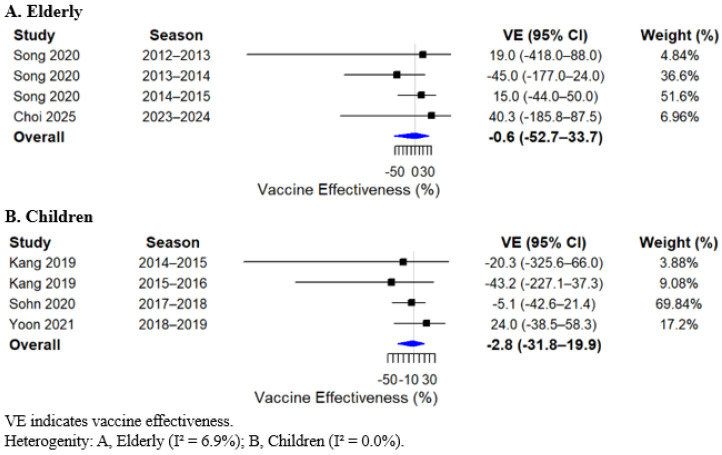
Forest plot of pooled vaccine effectiveness against influenza B by age group [12,14,16,17,20].

In the meta-analysis, statistically significant pooled VE estimates were identified across specific age groups and influenza virus types. Among adults, VE was 23.6% (95% CI, 13.8 to 32.2) (Figure 2A), and in children, 25.2% (95% CI, 8.2 to 39.0) (Figure 2C). When stratified by influenza virus type, the pooled VE against type A influenza among children was 32.4% (95% CI, 17.7 to 44.4) (Figure 3C). In contrast, no significant pooled VE estimates were observed in other age groups or for influenza type B and influenza A (H3N2). VE estimates in the elderly population also did not reach statistical significance; however, the point estimates were consistently lower across analyses. The pooled overall VE in the elderly was 8.3% (95% CI, −9.2 to 22.9). When stratified by virus type, VE in the elderly was 6.7% (95% CI, −14.0 to 23.7) for influenza A, −0.6% (95% CI, −52.7 to 33.7) for influenza B, and −21.4% (95% CI, −109.3 to 29.6) for influenza A (H3N2) (Figure 2, Figure 3 and Figure 4; Supplementary Figure S1).

### 3.3. Results of Publication Bias Assessment

Visual inspection of funnel plots did not reveal clear asymmetry (Supplementary Figure S2), although interpretation was limited by the small number of studies. In line with this, Egger’s test results were non-significant for all analyses (*p* > 0.05), except for influenza A in adults (*p* = 0.0342), indicating no strong evidence of publication bias overall.

### 3.4. Quality Assessment

Across the seven ROBINS-I domains, most studies demonstrated low to moderate levels of bias. All included studies were assessed as having low risk in Domain 4 (deviations from intended interventions), Domain 5 (missing data), and Domain 7 (selective reporting). Moderate risk of bias was frequently observed in participant selection (Domain 2) and outcome measurement (Domain 6). One study [14] was rated as having a serious risk due to potential confounding. Three studies [12,13,16] were rated as low risk across all domains. Taken together, the overall methodological quality of the included studies was considered acceptable (Supplementary Figure S3).

## 4. Discussion

This systematic review and meta-analysis included nine RWE studies evaluating influenza VE in Korea. Pooled estimates showed statistically significant VE in adults, children, and against influenza A in children. No significant VE was identified in other age groups or for influenza B or A (H3N2).

Based on this meta-analysis, statistically significant VE estimates were observed in adults (23.6%; 95% CI, 13.8 to 32.2) and children (25.2%; 95% CI, 8.2 to 39.0). When stratified by virus type, statistically significant VE was identified only for influenza A among children (32.4%; 95% CI, 17.7 to 44.4). When compared with findings from other global studies, the VE estimates observed in our analysis appear comparatively lower [26,27]. A recent global systematic review and meta-analysis of TND studies conducted from 2017 to 2022 reported an overall VE of 41.1% (95% CI, 39.2 to 43.5), including VE estimates of 48.6% (95% CI, 44.7 to 52.2) among children younger than 18 years and 49.1% (95% CI, 44.6 to 53.3) for influenza A [26]. Similarly, another global meta-analysis of studies published between 2013 and 2023, incorporating both TND and RCT designs, found that in TND studies alone, trivalent inactivated vaccines demonstrated VE of 43.8% (95% CI, 41.7 to 54.2) in strain-matched seasons and 40.1% (95% CI, 29.1 to 49.4) in mismatch seasons, while quadrivalent vaccines showed an overall VE of 34.3% (95% CI, 29.6 to 38.7) [27]. These differences suggest potential contextual or epidemiologic factors that may influence VE estimates at the national level.

Several factors may contribute to the comparatively lower VE observed in Korea. First, antigenic mismatch between vaccine strains and circulating viruses is a well-recognized determinant of diminished VE. In Korea, a 10-year evaluation (2011–2021) found concordance between WHO-recommended strains and predominant circulating strains in only three influenza seasons, indicating that frequent mismatch likely contributed to reduced VE [13]. Given that WHO strain recommendations are based on hemispheric trends, and regional variation in circulating viruses exists, Choi et al. (2024b) [13] emphasized the need for geographically tailored strain selection to improve national-level VE. Second, antigenic alterations associated with egg-based vaccine production may further reduce VE. Of the nine inactivated vaccines included in Korea’s NIP, eight are egg-based [10], suggesting that most vaccine recipients during the study period likely received egg-based formulations. Egg-adaptive changes arising during viral propagation in eggs can modify antigenic properties, thereby reducing the ability of vaccine-induced antibodies to neutralize circulating viruses [28]. In contrast, cell-based influenza vaccines avoid egg adaptation and have demonstrated greater antigenic similarity to circulating viruses [29]. Supporting laboratory evidence has shown that serial egg passage of influenza A (H3N2) viruses can induce amino acid substitutions in hemagglutinin, whereas cell-based propagation maintains antigenic stability even after repeated passages [30]. Real-world data have demonstrated a platform-specific effectiveness advantage. In the United States (US), cell-based quadrivalent vaccines showed approximately 10–14.8% higher relative VE than egg-based quadrivalent vaccines across the 2017–2020 seasons [31]. Similarly, a US-based systematic review and meta-analysis of studies published through 2021 reported an 11% (95% CI, 8 to 14) higher relative VE of cell-based vaccines during the 2017–2018 season [32].

Recent updates in global immunization guidelines reflect an expanding evidence base highlighting differences among influenza vaccine manufacturing platforms. The United Kingdom’s Joint Committee on Vaccination and Immunization (JCVI) has concluded that the cell-based inactivated influenza vaccine (IIVc) provides effectiveness comparable to that of adjuvanted, high-dose, and recombinant influenza vaccines in adults aged ≥ 65 years and includes IIVc among the preferred vaccine options for this age group. In addition, JCVI recommends IIVc as the preferred vaccine for most age groups other than adults aged ≥ 65 years—including children aged 6 months to <2 years in a risk group, children aged 2–18 years with contraindications to the live attenuated influenza vaccine, and at-risk adults aged 18–64 years, including pregnant women [33]. By contrast, the US Advisory Committee on Immunization Practices continues to preferentially recommend high-dose, adjuvanted, or recombinant influenza vaccines for adults aged ≥ 65 years without assigning equivalent priority to cell-based vaccines in this population [34]. These differing recommendations illustrate evolving but not yet fully aligned global perspectives regarding the relative performance of vaccine platforms. In Korea, current immunization guidelines do not differentiate preferred influenza vaccines by manufacturing platform, and cell-based vaccines are mentioned only as an option for individuals with a history of severe egg allergy or anaphylaxis [7]. Given the accumulating international evidence and policy developments, further evaluation of the role of cell-based influenza vaccines within Korea’s NIP may be warranted.

This study has several limitations. First, because the included studies were based on real-world data, unmeasured confounding may have influenced VE estimates. Second, key determinants of VE—such as antigenic match between vaccine and circulating strains and vaccine manufacturing platform—were not consistently reported across studies and therefore could not be fully incorporated into the analysis. Third, the small number of eligible studies and heterogeneity in study characteristics—including adjustment variables and population structure, and the gradual expansion of NIP target groups over time—may have reduced the precision of pooled estimates, particularly in subgroup analyses. Finally, although statistical tests did not suggest substantial publication bias, the limited number of included studies and the inability to account for unpublished data mean that the possibility of overestimation cannot be entirely excluded.

Despite these limitations, this study has notable strengths. First, it synthesizes real-world data-based influenza VE studies conducted in Korea over the past decade, integrating previously fragmented evidence across multiple seasons and organizing VE estimates by age group and virus type. This yields a clearer and more cohesive understanding of influenza VE within the Korean population. Second, by restricting the quantitative analysis to laboratory-confirmed influenza cases, the study enhances diagnostic accuracy and strengthens the validity of VE estimates, enabling more reliable assessment of vaccine performance in real-world settings.

## 5. Conclusions

This study synthesized a decade of fragmented influenza VE evidence from Korea, providing pooled estimates across age groups and virus types. Statistically significant VE was identified in the overall pooled analyses of adults and children, with effectiveness estimates lower than those reported globally. This may be related to antigenic mismatch between vaccine and circulating strains and antigenic changes associated with egg-based vaccine production. As global immunization guidelines evolve in response to emerging evidence, further evaluation of the role of alternative vaccine platforms, including cell-based vaccines, may be warranted within Korea’s national immunization strategy.

## Figures and Tables

**Table 1:** Selected characteristics of included studies for both qualitative and quantitative analyses (n = 9).

Study	Setting/Design	Population	Seasons	Influenza Diagnosis Criteria	Vaccine Type	Total N (M:F)	VE (95% CI)
Noh 2017 [18]	Hospital/TND	Patient over 19 years (1) ER with ILI (2) Hospitalized with ILI	2016–2017	(1) RDT (2) PCR	Seasonal	400 (162:238)	(1) Influenza A A. Overall: −35.6 (−114.7 to 14.3) B. 19–64 years: −67.1 (−202.9 to 7.9) C. ≥65 years: 22.7 (−71.8 to 65.2) (2) Influenza A (H3N2) A. Overall: −52.1 (−147.2 to 6.4) B. 19–64 years: −70.0 (−212.0 to 7.4) C. ≥65 years: 4.3 (−137.8 to 61.5)
Kang 2019 [14]	Hospital, Health center/Cohort	Children aged 6 months to <5 years with ILI	2014–2016	(1) RDT (2) PCR	Seasonal	(1) 2014–2015: 568 (307:261) (2) 2015–2016: 818 (436:382)	(1) 2014–2015 A. Overall: 38.4 (−34.6 to 71.8) B. Influenza A: 50.7 (−38.9 to 82.5) (2) 2015–2016 A. Overall: 23.8 (−20.2 to 51.6) B. Influenza A: 48.5 (6.2 to 71.8)
Kim 2020 [15]	School survey/Cross-sectional	Children 8–13 years	2016–2017	Survey, self-reported	Seasonal	(1) Vaccinated: 1696 (815:881) (2) Unvaccinated: 998 (494:504)	Overall: 3.6 (−12.1 to 17.0) (1) 8 years: −2.8 (−49.5 to 29.4) (2) 9 years: 12.1 (−25.8 to 38.6) (3) 10 years: 20.5 (−11.0 to 43.0) (4) 11 years: 4.7 (−39.8 to 35.1) (5) 12 years: −5.6 (−53.7 to 27.5) (6) 13 years: −7.6 (−61.3 to 28.2)
Sohn 2020 [16]	Hospital/TND	Children aged 6 to 59 months with ILI	2017–2018	(1) RDT (2) PCR	Seasonal	4738 (2595:2143)	(1) PCR A. Overall: 53.4 (25.3 to 70.5) - 6–35 months: 44.1 (−0.2 to 67.8) - 36–59 months: 59.3 (8.8 to 81.9) B. Influenza A: 68.8 (38.7 to 84.1) C. Influenza B: 29.7 (−35.1 to 61.8) (2) RDT A. Overall: 14.8 (−4.4 to 30.3) - 6–35 months: 14.3 (−10.9 to 33.3) - 36–59 months: −3.4 (−41.4 to 23.9) B. Influenza A: 24.2 (3.1 to 40.2) C. Influenza B: −5.1 (−42.6 to 21.4)
Song 2020 [12]	Hospital/TND	Patient over 65 years with ILI	2012–2015	(1) RDT (2) PCR	Seasonal	(1) 2012–2023: 510 (251:259) (2) 2013–2014: 604 (308:296) (3) 2014–2015: 1039 (532:507)	(1) 2012–2023 A. Influenza A: 34.0 (−1.0 to 60.0) B. Influenza B: 19.0 (−418.0 to 88.0) (2) 2013–2014 A. Influenza A: −25.0 (−96.0 to 20.0) B. Influenza B: −45.0 (−177.0 to 24.0) (3) 2014–2015 A. Influenza A: −22.0 (−71.0 to 13.0) B. Influenza B: 15.0 (−44.0 to 50.0)
Yoon 2021 [17]	Hospital (Emergency Department)/TND	Children aged from 6 months to 12 years with ILI	2018–2019	RDT	Seasonal	1417 (732:685)	(1) Overall: 36.4 (13.9 to 53.1) A. 6 months−12 years: 35.6 (10.5 to 53.7) B. 13–18 years: 37.4 (−49.7 to 73.8) (2) Influenza A: 37.6 (12.6 to 55.5) (3) Influenza B: 24.0 (−38.5 to 58.3)
Choi 2024 [19]	Hospital/TND	Patient over 19 years (1) ER or Outpatient with ILI (2) Inpatient with ILI or laboratory-confirmed influenza	2023–2024	(1) RDT (2) PCR	Seasonal	2632 (1135:1497)	(1) Overall: 22.5 (6.6 to 35.8) A. 19–64 years: 24.3 (5.3 to 39.5) B. ≥65 years: 17.4 (−17.1 to 41.8) (2) Influenza A: 22.3 (6.1 to 35.7) A. 19–64 years: 23.9 (4.5 to 39.3) B. ≥65 years: 17.4 (−17.1 to 41.8)
Choi 2024 [13]	Hospital/TND	Patient over 65 years (1) ER or outpatient with ILI (2) Inpatient with ILI or laboratory-confirmed influenza	2011–2021	RDT	Seasonal	5322 (2690:2632)	2011–2021: 2.9 (−10.6 to 14.7) (1) 2011–2012: −46.9 (−127.6 to 5.2) (2) 2012–2013: 10.5 (−42.4 to 43.7) (3) 2013–2014: 25.6 (−8.5 to 49.0) (4) 2014–2015: 9.2 (−24.5 to 33.7) (5) 2015–2016: −35.6 (−86.2 to 1.2) (6) 2016–2017: 47.7 (22.6 to 64.7) (7) 2017–2018: 6.9 (−40.9 to 38.4) (8) 2018–2019: −5.4 (−116.9 to 48.8) (9) 2019–2020: 3.0 (−83.9 to 48.8) (10) 2020–2021: N/A
Choi 2025 [20]	Hospital/TND	Patient over 19 years (1) ER or outpatient with ILI (2) Inpatient with ILI or laboratory-confirmed influenza	2023–2024	(1) RDT (2) PCR	Seasonal	3390 (1440:1950)	(1) Overall: 24.3 (11.5 to 35.2) A. 19–49 years: 31.1 (15.7 to 43.7) B. 50–64 years: 10.9 (−36.3 to 41.8) C. ≥65 years: 13.5 (−17.9 to 36.6) (2) Influenza A: 19.6 (5.7 to 31.4) A. 19–49 years: 23.8 (6.1 to 38.2) B. 50–64 years: 12.2 (−35.3 to 43.0) C. ≥65 years: 13.1 (−18.7 to 36.3) (3) Influenza A (H1N1): 36.5 (−8.9 to 63) A. 19–49 years: N/A B. 50–64 years: −86.8 (−506.5 to 42.5) C. ≥65 years: 46.9 (2.5 to 71.1) (4) Influenza A (H3N2): 13.2 (−97.4 to 20.8) A. 19–49 years: 49.5 (−139.0 to 89.3) B. 50–64 years: N/A C. ≥65 years: −38.6 (−173.4 to 29.8) (5) Influenza B: 60.1 (39.4 to 73.8) A. 19–49 years: 64.8 (43.5 to 78.1) B. 50–64 years: 27.0 (−178.9 to 80.9) C. ≥65 years: 40.3 (−185.8 to 87.5)

TND, test-negative design; ER, emergency room; ILI, influenza-like illness; RDT, rapid diagnostic test (rapid antigen-based test for influenza; terminology such as “RIDT,” “RAT,” and “influenza rapid test” used in the original studies was harmonized as RDT; PCR, polymerase chain reaction (terminology used in the original studies, including “real-time PCR,” was standardized as PCR); M, male; F, female; VE, vaccine effectiveness.

## Data Availability

All data analyzed in this study were derived from previously published studies and are available in the cited articles and Supplementary Materials.

## Supplementary Materials

The following supporting information can be downloaded at: https://www.mdpi.com/article/10.3390/vaccines14030217/s1, Figure S1. Forest plot of pooled vaccine effectiveness against influenza A (H3N2) by age group. Figure S2. Funnel plots for assessing publication bias. Figure S3. Risk of bias assessment of the included studies using the ROBINS-I tool. Table S1. Search Strategies used for each database; Table S2. PRISMA 2020 Checklist.
